# Immune regulation and organ damage link adiponectin to sepsis

**DOI:** 10.3389/fimmu.2024.1444884

**Published:** 2024-11-27

**Authors:** Lili Zhang, Yuning Lin, Zhongying Zhang, Yuting Chen, Jinqing Zhong

**Affiliations:** Medical Laboratory Center, Xiamen Humanity Hospital, Xiamen, Fujian, China

**Keywords:** adiponectin, sepsis, hyperinflammation, biomarker, immune cell

## Abstract

Sepsis is a life-threatening syndrome characterized by organ dysfunction, resulting from an uncontrolled or abnormal immune response to infection, which leads to septicemia. It involves a disruption of immune homeostasis, marked by the release of Inflammatory factors and dysfunction of immune cells. Adiponectin is widely recognized as an anti-inflammatory mediator, playing a crucial role in regulating immune cell function and exerting protective effects on tissues and organs. However, the physiological role of adiponectin in septicemia remains unclear due to the condition’s association with immune response dysregulation and organ damage. This study focuses on the potential relationship between adiponectin and excessive immune responses, along with organ injury in septicemia. Additionally, we investigate possible explanations for the observed discrepancies in adiponectin levels among critically ill or deceased patients compared to theoretical expectations, aiming to provide valuable insights for clinical diagnostics and therapeutic interventions in sepsis.

## 1 Introduction

Septicemia is a syndrome of physiological, pathological, and biochemical abnormalities caused by infection, which can lead to dysfunction in multiple organs. At the cellular and molecular levels, the pathogenesis of septicemia involves an imbalance in the systemic inflammatory response or immune dysregulation (1). Inflammatory imbalance runs throughout the course of septicemia and is the most critical underlying factor in its onset. Pathogens, including bacteria, fungi, parasites, and viruses, can trigger an inflammatory imbalance in the host, leading to the development of septicemia (2). During sepsis, the immune system is activated by pathogen-associated and host-derived molecular patterns. The initial acute response typically activates the host’s innate immune system, leading macrophages, monocytes, and dendritic cells to produce a series of cytokines. Subsequently, when inflammatory cell infiltration occurs, regulatory cell functions are suppressed, and high levels of pro-inflammatory cytokines overwhelm the low levels of anti-inflammatory cytokines, a cytokine storm may occur (3). This cytokine storm further disrupts endothelial cells, leading to coagulation dysfunction and vascular leakage (4), ultimately resulting in a series of severe complications associated with septicemia.

Adiponectin is produced by adipocytes and has been shown to play an important role in inflammation and immune regulation. Research indicates that serum adiponectin levels change during different stages of sepsis, with levels decreasing in infected patients who later develop sepsis. The relationship between adiponectin and the severity and outcomes of sepsis remains unclear. It is uncertain whether this phenomenon arises from the disease process itself or if patients with lower hormone levels are more likely to experience an increased inflammatory response, making them more susceptible to sepsis. The mechanisms by which adiponectin exerts its immunoregulatory and protective effects seem to be related to sepsis. Therefore, we focus on the potential mechanisms of adiponectin in sepsis, including its regulation of innate and adaptive immune cell responses and its protective effects on organs. Some studies also suggest that patients with septic shock or those who have died exhibit higher levels of adiponectin, which appears to contradict the theory that adiponectin has anti-inflammatory and protective effects on the body. This review also explores possible reasons for this discrepancy.

## 2 Structure and receptors of adiponectin

**Figure 1:**
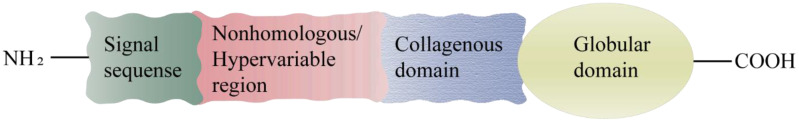
Adiponectin monomeric structure.

**Figure 2:**
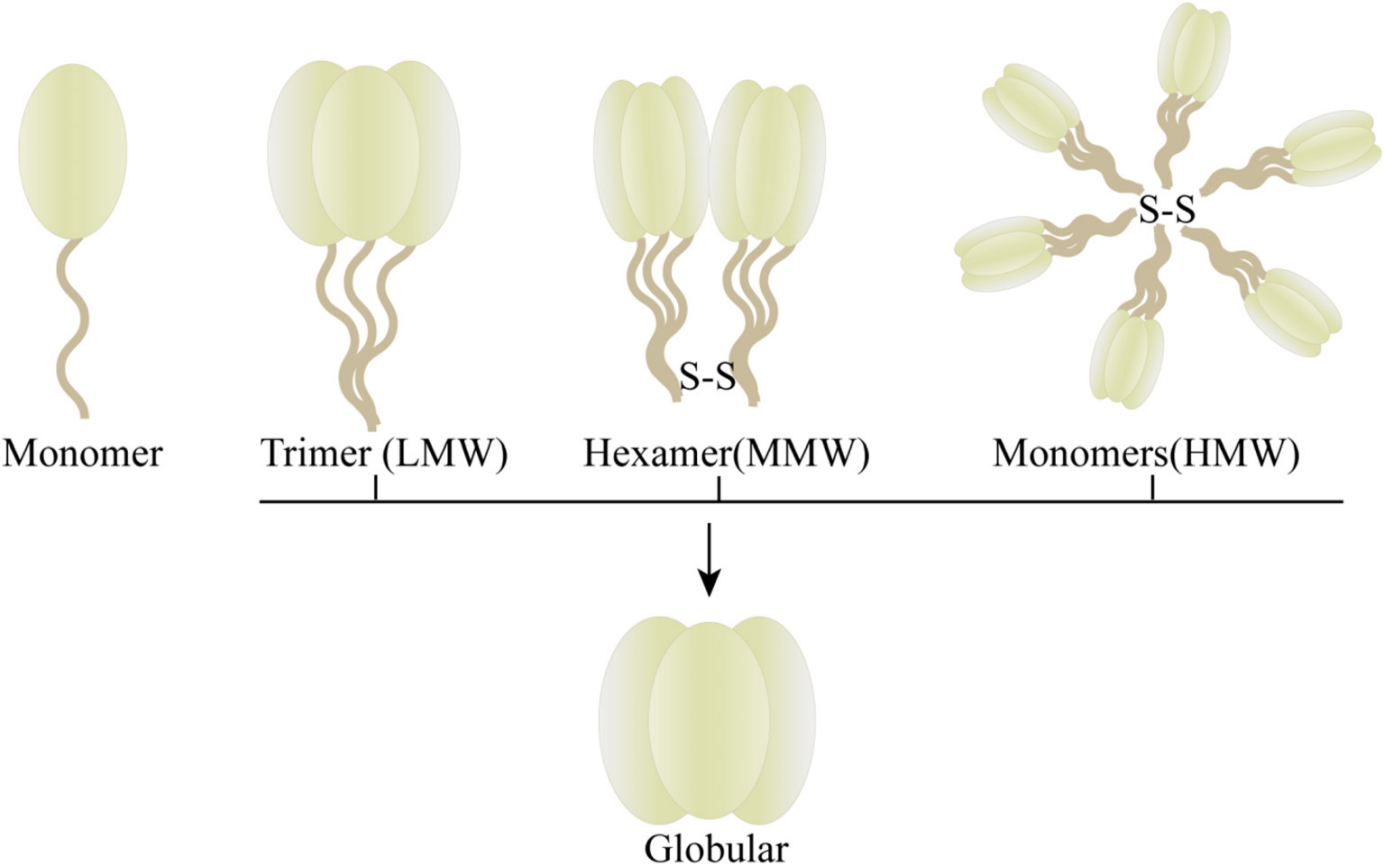
Form of adiponectin with different molecular weight.

The protein adiponectin, synthesized and secreted by adipocytes, is comprised of a monomer with an approximate molecular weight of 30KDa. This monomeric structure consists of an N-terminal signal sequence, a nonhomologous or hypervariable region, a collagenous domain, and a C1q-like globular domain (**Figure 1**) (5). The full-length adiponectin exists in three distinct forms with varying molecular weights (**Figure 2**): high molecular weight form (HMW, 12-18 monomers, ∼360-540kDa), medium molecular weight form (MMW, hexamer, ∼180 kDa) and low molecular weight form (LMW, trimer, ∼90 kDa). Oligomers formed by different molecular weights exhibit diverse bioactivities, with HMW adiponectin demonstrating the highest level of biological activity (5–8). Full-length adiponectin can undergo proteolytic cleavage, resulting in hydrolyzed fragments that correspond to globular adiponectin, which also exhibits biological activity (7). There are three distinct types of adiponectin receptors, namely AdipoR1, AdipoR2, and T-cadherin, which exhibit widespread distribution in various tissues and organs throughout the human body. AdipoR1 is predominantly abundant in skeletal muscle tissue, while AdipoR2 is primarily expressed in the liver. T-cadherin demonstrates a broad distribution across human tissues and organs with notable expression levels observed within the cardiovascular system but relatively lower expression levels within muscular tissues (6, 9, 10). The binding specificity of AdipoR1 and AdipoR2 towards adiponectin differs, with AdipoR1 displaying a higher affinity for globular forms, while AdipoR2 demonstrates equivalent affinities for both globular and full-length variants (7). T-cadherin serves as a receptor for both MMW adiponectin and HMW adiponectin (6). The physiological functions and protective effects of adiponectin are exerted by modulating diverse signaling pathways through the activation of AdipoR1 and AdipoR2 (9, 10). The formation of a complex between adiponectin and T-cadherin promotes exosome generation and secretion, thus facilitating cellular expulsion of cytotoxic substances. Adiponectin, along with its interaction with T-cadherin, plays a crucial role in preserving systemic homeostasis (11).

The majority of immune cells express adiponectin receptors on their surface (6), and the anti-inflammatory effect of adiponectin is mediated through its binding to these receptors. In recent years, numerous studies have elucidated the regulatory role of adiponectin in modulating inflammation (12–14). Adiponectin may play a role in the pathogenesis of systemic inflammatory response in sepsis, encompassing direct effects on inflammatory cells and interactions with inflammatory cytokines, cellular function, cell injury, and other inflammation-related pathways. Research has demonstrated that adiponectin can attenuate the phagocytic activity of lipopolysaccharide (LPS)-stimulated macrophages and inhibit the production of inflammatory cytokines (15).

## 3 Association of adiponectin with the immune response to sepsis

In sepsis, the immune response consisting of the innate and adaptive immune systems plays a key role. The innate immune system is the first line of defense against pathogens. It is not specific and can quickly play a role in anti-infection. Once activated, innate immune cells go on to produce and release a large number of inflammatory mediators (16). The adaptive immune system consists of T and B lymphocytes, involves specific antigen-specific responses to pathogens, and is regulated by interactions of innate immune cells. Both the innate and adaptive immune responses undergo dramatic changes when infectious injury occurs (17). Although the role of adiponectin in the immune system has not been fully determined, it plays an important role in regulating innate and adaptive immune cell function and may have a general anti-inflammatory effect on innate immune cells (18).

Data from animal studies indicate that serum adiponectin is lower in dogs with sepsis compared with dogs with low-grade systemic inflammation (LGSI) (19). Salivary adiponectin concentrations were statistically significantly lower in septic dogs than in healthy dogs (20). Early Ingeborg D Welters et al. conducted a prospective observational pilot study in 21 septic patients, analyzing samples for total adiponectin, HMW adiponectin, and HMW/total adiponectin ratio. The data showed that both HMW adiponectin and total adiponectin were increased compared with admission in patients with clinical recovery from sepsis (21). In the clinical study by Andreas Hillenbrand et al., the median plasma adiponectin level 1 day after a sepsis episode was slightly lower than that before a sepsis episode (22).

In these studies, the mechanism of the change of adiponectin level is not mentioned, but it suggests that in the process of inflammation to sepsis, the decrease of adiponectin may cause transitional inflammation and lead to sepsis. The increase of adiponectin may restore immune homeostasis and contribute to the recovery of patients with sepsis. Therefore, adiponectin, as an anti-inflammatory factor, may play a potentially important role in the process of severe inflammation to sepsis.

## 4 Adiponectin regulates immune cells to prevent hyperinflammation

Hyperinflammation, which occurs when the body overreacts to pathogens, is a central stage in the early pathogenesis of sepsis (23, 24). Hyperinflammation results from uncontrolled activity of proinflammatory effector mechanisms, including activated immune cells, accompanied by dysregulation of aerobic or nitrogen free radicals and cytokine production, as well as activation of the complement and coagulation systems (25). These uncontrolled activities can cause collateral damage and promote the pathogenesis of sepsis. Adiponectin can reduce the intense inflammatory effects of the body by regulating the immune cells (18).

### 4.1 Adiponectin regulates innate immune cells

#### 4.1.1 Macrophages

As an important defense cell of the immune system, macrophages play a crucial role in both innate and acquired immunity. Activating macrophages to secrete proinflammatory mediators is a necessary response to defend against pathogens (26). LPS is a major mediator of sepsis during Gram-negative bacterial infections and other acute infectious diseases (27). In sepsis, macrophages release tumor necrosis factor-α (TNF-α) simultaneously in various organs under the induction of LPS, leading to multiple organ damage (28). Studies have shown that adiponectin can inhibit LPS-induced TNF-α gene transcription in macrophages and negatively regulate inflammatory response by inhibiting the proliferation of macrophage precursor cells or inhibiting the function of mature macrophages (29, 30). Thus, in the study of the effect of adiponectin on sepsis, macrophages have attracted much attention.

AdipoR1, AdipoR2 and T-cadherin are present in monocytes/macrophages, and adiponectin may regulate macrophage proliferation and function through a receptor-mediated mechanism (31). It can affect the macrophage response by promoting the secretion of macrophage anti-inflammatory cytokines, thereby contributing to the resolution of inflammation. For example, studies have shown that adiponectin can bind to T-cadherin, activate the PI4K pathway, promote the proliferation of anti-inflammatory macrophages, or inhibit the production of pro-inflammatory mediators by blocking the signaling pathway of pro-inflammatory cytokine secretion (32). Hélène Salvator et al. showed that the expression of adiponectin receptors AdipoR1 and AdipoR2 in human lung macrophages, Low molecular weight adiponectin and adiponectin receptor agonist (AdipoRon) significantly inhibited LPS and poly in a concentration dependent manner (I:C) -induced release of tumor necrosis factor-α, IL-6, and chemokines (CCL3, CCL4, CCL5, CXCL1, CXCL8, CXCL10) and IL-4-induced chemokines (CCL13, CCL17, CCL22) (30). It has also been suggested that adiponectin, which shares a clear sequence similarity with complement C1q, regulates the phagocytic activity of macrophages through C1qRp (29).

**Figure 3:**
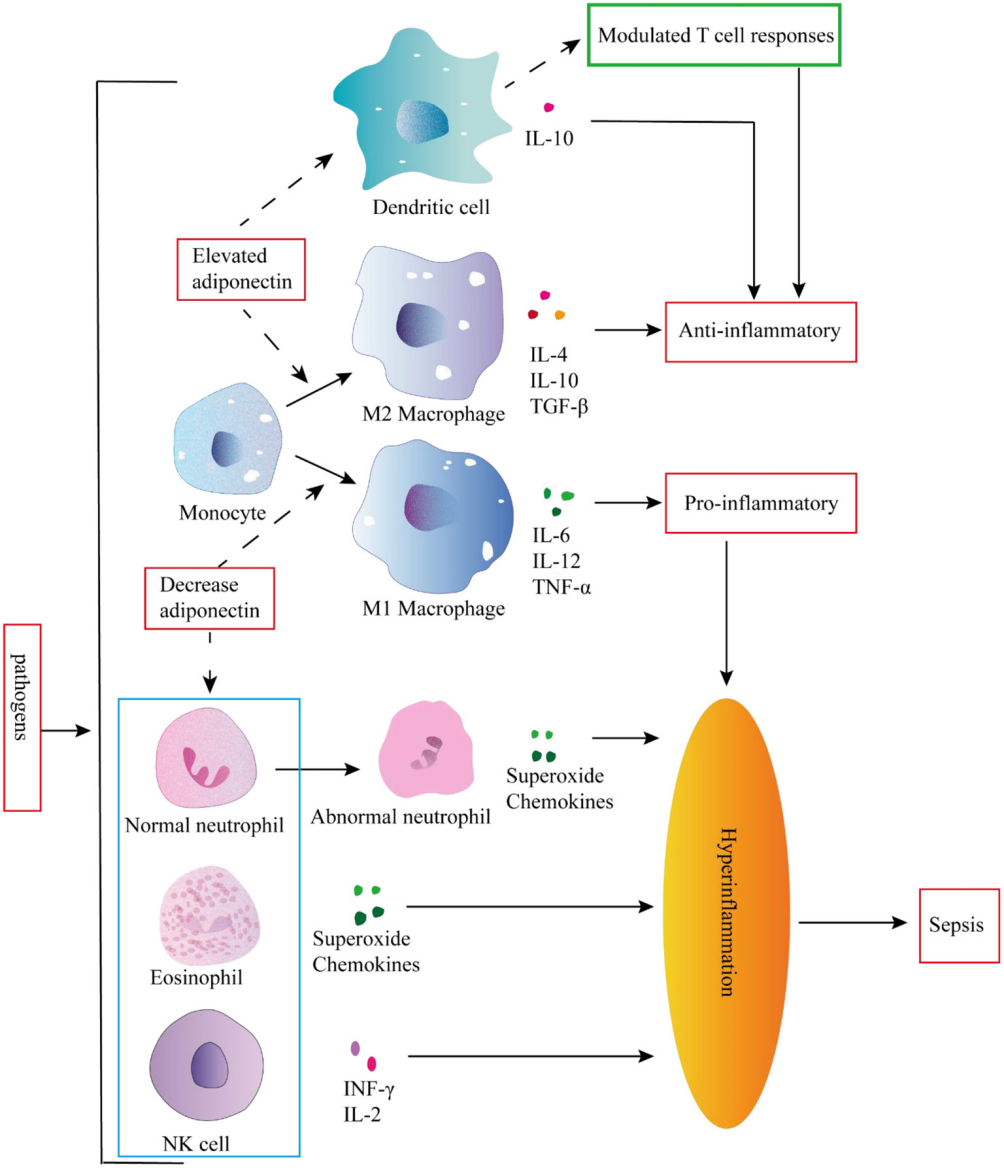
Inflammation occurs in response to pathogen invasion. Inflammation is a response triggered by pathogen invasion. Elevated adiponectin can attenuate the secretion of pro-inflammatory cytokines and enhance the secretion of anti-inflammatory cytokines in dendritic cells (DCs). Conversely, adiponectin can modulate DC-mediated T cell responses to exert an anti-inflammatory role. In addition elevated adiponectin can stimulate monocytes to differentiate into M2 macrophages, whereas decreased adiponectin can promote monocyte differentiation into M1 macrophages, and the pro-inflammatory activities of granulocytes and NK cells could not be controlled, leading to hyperinflammation and subsequent sepsis.

The development of sepsis is closely associated with uncontrolled systemic inflammation. Dysregulation of M1 macrophage polarization plays a pivotal role in driving severe inflammation. Adiponectin facilitates the alternative activation of human monocytes into anti-inflammatory M2 macrophages, contrasting with the classically activated M1 phenotype (33). Adiponectin primarily acts through AdipoR2 to induce polarization of macrophages towards an M2 phenotype, significantly enhancing the expression of various markers associated with M2 macrophages, such as Arg-1, scavenger receptors (CD36), surface lectins (macrophage galactose N-acetylgalactosamine-specific lectins 1 and 2 [Mgl1 and Mgl2)], secreted lectins [chitinase-3-like 3 (Chi3l3 or YM-1)], and cytokine receptors [IL-4 receptor α (IL-4Rα)] (34) (**Figure 3**). In addition, M2 macrophages can inhibit inflammation, induce the secretion of anti-inflammatory cytokines such as IL-10 and TGF-β, promote the removal of apoptotic cell debris and tissue repair (35, 36). The M1 macrophages, in contrast, play a pivotal role in promoting inflammation and facilitating the production of proinflammatory cytokines (such as IL-6, TNF-α, and IL-12) as well as reactive oxygen species. These factors further contribute to monocyte recruitment and lipid oxidation, ultimately leading to tissue damage (37).

#### 4.1.2 Granulocytes

Granulocytes, including neutrophils, eosinophils, and basophils, are the predominant circulating cells within the innate immune system. These circulating granulocytes, primarily neutrophils, possess the ability to traverse the endothelial barrier and initiate diverse effector mechanisms in order to combat invasive pathogens. Eosinophils and basophils also play a crucial role in allergic reactions and defense against parasites. Moreover, granulocytes actively regulate immune responses, wound healing, and tissue repair through the release of various cytokines and lipid mediators (38).

Neutrophils are abundant, short-lived leukocytes that play a pivotal role in the immune defense against microbial infections. Under physiological conditions, neutrophils undergo apoptosis to maintain their homeostasis. However, during sepsis, neutrophils experience several functional alterations, including diminished migration capacity, altered antimicrobial activity, and delayed apoptosis, thereby contributing to immune dysfunction and persistent inflammation (39–42). The changes in neutrophils contribute to the progression of inflammation towards sepsis and the development of secondary complications. Adiponectin inhibits the apoptosis of neutrophils with abnormal morphology and function by activating AMPK, which enables healthy neutrophils to quickly remove pro-inflammatory factors and exert anti-inflammatory effects (43). Adiponectin also exerts suppressive effects on the production of CXCL8 by neutrophils (44), inhibits O2-* generation, potentially through the regulation of NADPH oxidase (45), and suppresses NET release by suppressing ROS production (46). Collectively, deficiency in adiponectin may lead to altered neutrophil function and increase susceptibility to sepsis (**Figure 3**).

Eosinophils possess receptors for many inflammatory mediators and are capable of producing and releasing a diverse range of biologically active molecules, including cytotoxic proteins, lipid mediators, chemokines, and cytokines (47). Eosinophils play a crucial role in modulating both local and systemic immune responses (48). The findings of Nansalmaa Amarsaikhan et al. suggest that adiponectin plays a role in inhibiting excessive lung inflammation in invasive aspergillosis. Additionally, it was observed that eosinophil recruitment and activation are increased in mice lacking adiponectin (49). These results imply that adiponectin potentially regulates the anti-inflammatory impact of eosinophils. Nonetheless, a systematic review and meta-analysis revealed only a moderate correlation between eosinopenia and sepsis, thus indicating limited clinical utility of eosinopenia as a diagnostic tool for sepsis (50). Although the prognostic significance of basophils, a type of innate immune cells, has been established in sepsis patients (51), the role of adiponectin in modulating basophil function remains unexplored.

#### 4.1.3 NK cell

During early sepsis, NK cells activation is dysregulated and secretes large amounts of cytokines, contributing to a positive feedback loop and amplifying the pro-inflammatory cytokine storm (52). NK cells are a key component of innate immune systems, and their activity is regulated by adiponectin. The production of IFN-gamma, one of the NF-kappaB target genes in NK cells, was also found to be suppressed by adiponectin, accompanied by the subsequent down-regulation of IFN-gamma-inducible TRAIL and Fas ligand expression. Adiponectin is a potent negative regulator of IL-2-induced NK cell activation (53). Therefore, adiponectin may function as a regulatory factor in Hyperinflammation in NK cells, thereby exerting an anti-inflammatory effect *in vivo* (**Figure 3**).

#### 4.1.4 Dendritic cell

Dendritic cells (DCs) are antigen-presenting cells that orchestrate both innate and adaptive immune responses. DCs not only recognize pathogens and danger signals through pattern recognition receptors (PRRs), triggering intracellular signaling cascades, releasing antimicrobial mediators, and initiating innate immune responses, but also efficiently capture, process, and present antigens to T lymphocytes in order to facilitate the activation of adaptive immune responses (54, 55). The activation of DCs by diverse PRRs induces distinct patterns of cytokine/inflammatory mediator secretion which subsequently determine the nature of the T cell response (56).

The expression of adiponectin receptors AdipoR1 and AdipoR2 is observed in DCs. AdipoR1 activates the AMPK and MAPKp38 pathways, leading to the stimulation of IL10 production. On the other hand, AdipoR2 modulates the inflammatory process by activating COX-2 and PPARγ pathways. Activation of these signaling cascades effectively inhibits NF-κB activation in DCs, thereby attenuating their ability to mediate antigen-specific T cell responses (57). Julia Yuen Shan Tsang et al. discovered that co-culturing adiponectin conditioned DCs (ADN-DCs) with T cells *in vitro* resulted in reduced T cell proliferation and IL-2 production, while exhibiting an increased proportion of CD4^+^ CD25^+^ Foxp3^+^ regulatory T cells (Tregs) within the T cell and ADN-DCs co-cultures (58). These findings suggest that the immunomodulatory effects of adiponectin on immune responses may be mediated by modulating DCs function to exert anti-inflammatory effects. On one hand, it can attenuate the secretion of pro-inflammatory factors and enhance the production of anti-inflammatory factors by DCs; on the other hand, it can alter DCs-mediated T cell response (**Figure 3**).

In conclusion, adiponectin is a key regulator of the innate immune system and plays an important role in the progression of inflammation to sepsis progression. It may regulate metabolism by targeting the innate immune system under physiological and pathological conditions, and determining the mechanism of action of adiponectin in regulating innate immunity will be critical for adiponectin-based therapeutic interventions.

### 4.2 Adiponectin regulates adaptive immune cells

**Figure 4:**
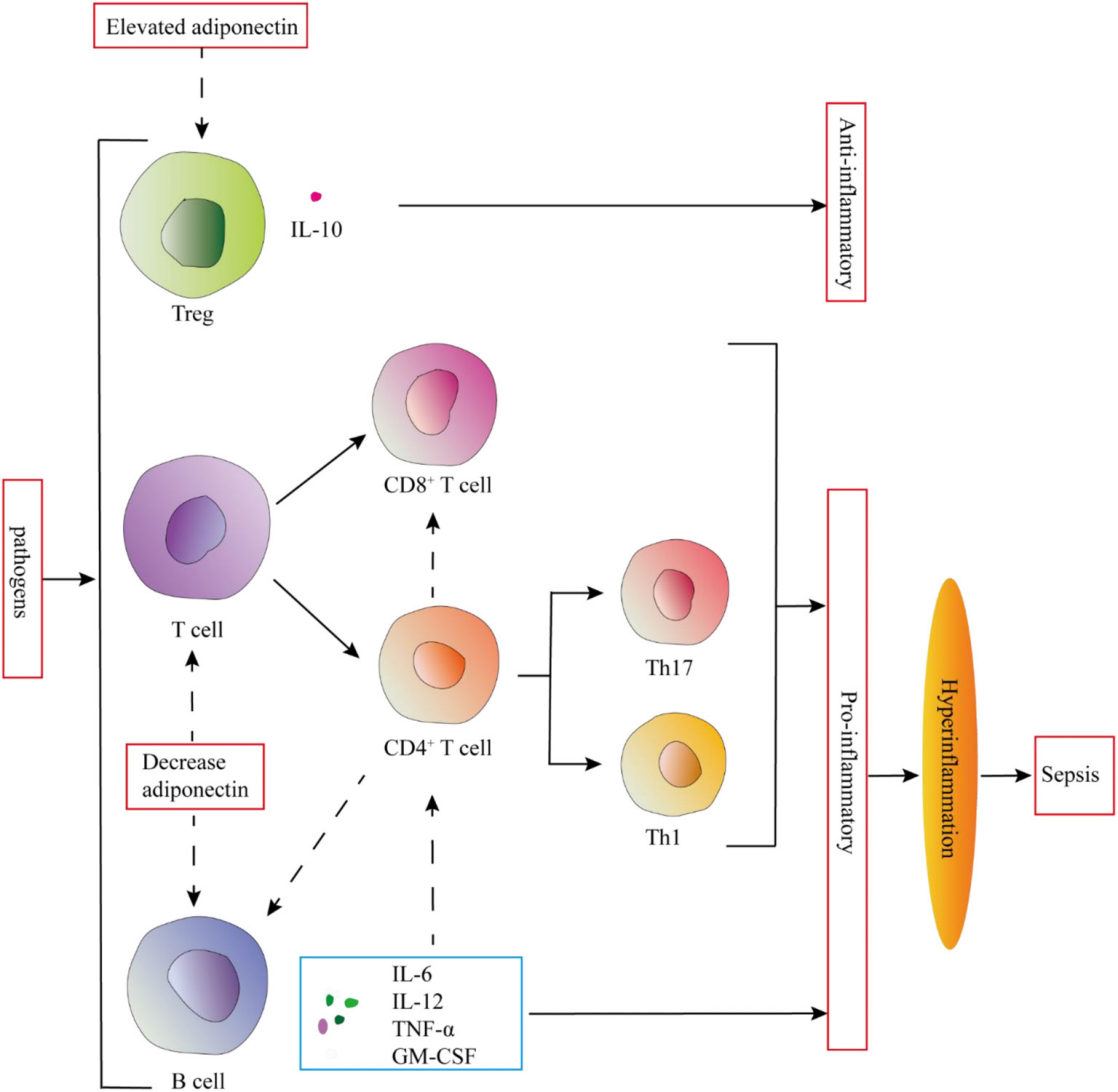
Adiponectin may regulate the excessive inflammatory response in sepsis by regulating the proliferation, differentiation, and function of T and B lymphocytes. Elevated adiponectin levels promote IL-10 secretion by Tregs, while also suppressing the proliferation, differentiation, and function of T cells. Furthermore, it inhibits the secretion of proinflammatory cytokines by B cells. These adaptive immune modulations mediated by adiponectin serve to prevent the onset of hyperinflammation.

Sepsis has significant effects on tissue and circulating T lymphocytes, particularly CD4^+^ T cells. CD4^+^ T helper (Th) cells play a vital role in the body’s immunological processes by assisting other cell types, such as B cells during differentiation, activating cytotoxic T cells, and stimulating monocytes. In sepsis, B cells can produce a variety of pro-inflammatory cytokines like IL-6, TNF, IL-3, and GM-CSF, which amplify the systemic inflammatory response. These cytokines also play an important role in polarizing CD4^+^ T cells towards Th1 and Th17 phenotypes and in activating innate immune cells such as monocytes (59). T and B lymphocytes both express adiponectin receptors. Thus, adiponectin may regulate the excessive inflammatory response in sepsis by regulating the proliferation, differentiation, and function of T and B lymphocytes (60) (**Figure 4**).

#### 4.2.1 T lymphocytes

This is consistent with the results observed in adiponectin-deficient mice, which showed a higher proportion of CD137^+^ T cells after infection with Coxsackie B virus, further indicating that adiponectin has a negative regulatory effect on T cells (60). When treated with adiponectin *in vitro*, the expansion of antigen-specific T cells is significantly reduced, and cytokine production decreases. This is primarily due to adiponectin promoting T cell apoptosis (60). In *in vitro* experiments, adiponectin inhibits TNF-α and IFN-γ-induced transendothelial migration of human peripheral blood lymphocytes in a dose-dependent manner, primarily affecting memory T cells (61). The globular domain of adiponectin inhibits T cell activation by interacting with the leukocyte-associated immunoglobulin-like receptor-1 (LAIR-1), thereby confirming the anti-inflammatory effect of adiponectin (62). Moreover, adiponectin inhibits the function and proliferation of CD8^+^ T cells, indicating that adiponectin negatively regulates the activity of cytotoxic T lymphocytes through its interaction with AdipoR1/R (60). The activation of CD8^+^ T cells triggers macrophage activation and induces a pro-inflammatory phenotypic shift (63).

Th1 and Th17 cells are pro-inflammatory T cell subsets that play a significant role in the pathogenesis of metabolic disorders (64). Recent studies have found that adiponectin can directly reduce the number of IFN-γ and IL-17 positive CD4^+^ T cells in high-fat diet mice and inhibit the differentiation of naive T cells into Th1 and Th17 cells (65). During the differentiation of naive T cells into Th cells, metabolic reprogramming occurs to meet their energy demands and to provide essential substrates for T cell proliferation and differentiation. Regulating these metabolic pathways can significantly influence the fate and function of T cells. Th17 cells primarily rely on aerobic glycolysis as their main energy source (66, 67). Adiponectin significantly reduces the glycolytic rate of Th17 cells, and this effect on Th17 glycolysis is mediated through the AMPK pathway. In contrast, the differentiation and glycolytic changes in Th1 cells are independent of AMPK (65). Exogenous adiponectin inhibits IFN-γ production during Th1 cell differentiation and downregulates Th17 cytokine expression during Th17 cell differentiation. This process is mediated through the SIRT1-PPARγ-RORγt pathway (68). Adiponectin inhibits the differentiation of Th1 and Th17 cells through AdipoR1, leading to the downregulation of CD80 and CD40. It also hinders the *in vitro* differentiation of Th1 and Th17 cells by suppressing antigen presentation (69). In obesity and insulin resistance (IR) conditions, the number of anti-inflammatory regulatory T cells (Tregs) in AT is significantly reduced.

New evidence suggests that globular adiponectin (g-APN) affects the ability of Tregs to produce IL-10 and induces the expression of Forkhead Box P3 (FOXP3) (58, 70). Zhang et al.’s study found that thymic Tregs expressing adiponectin may be involved in the differentiation of CD117^+^CD4^+^CD25^+^ precursor cells during the CD4^+^CD8^+^ double-positive (DP) stage in the thymus. This suggests that adiponectin plays a significant role in the selection and development of T cells within the thymus (71). AdipoR1 is expressed on Tregs residing in AT, and this expression is reduced in obesity (72). Furthermore, Tregs expressing AdipoR1 exhibit significantly elevated levels of IL-10, suggesting that the g-APN-AdipoR1 pathway may have a protective effect against inflammation. Ligand-induced upregulation of AdipoR1 expression could enhance the responsiveness of Tregs to adiponectin, promoting IL-10 production and potentially reducing inflammation (70).

In general, adiponectin has been shown to play a beneficial role in metabolic disorders by regulating T cell differentiation and function. Adiponectin can inhibit the differentiation of inflammatory Th1 and Th17 cells while enhancing the function of Tregs. Additionally, adiponectin can suppress T cell migration and proliferation, which may help reduce inflammation and improve metabolic profiles.

#### 4.2.2 B lymphocytes

B cells are not only capable of producing antibodies but also secrete pro-inflammatory or anti-inflammatory cytokines. In obesity, B cells secrete more pro-inflammatory cytokines, such as IL-6 and IFN-γ, while the secretion of anti-inflammatory cytokines is reduced (73). Studies have shown that adiponectin can inhibit B lymphopoiesis in long-term bone marrow cultures, suggesting that a decrease in adiponectin levels may promote B cell expansion (74). In contrast, recent studies have found that splenic B cells from normal mice express high levels of the AdipoR1 receptor, and stimulation by adiponectin significantly enhances B cell proliferation and their differentiation into plasma cells (75). Adiponectin upregulates the expression of Blimp-1 by activating the PI3K/Akt1/STAT3 pathway, indicating the crucial role of this signaling pathway in B cell differentiation responses (75). However, the specific mechanisms by which adiponectin regulates B cell function and its role in obesity modulation are not yet fully understood, and further research is needed to elucidate these processes.

In summary, a reduction in adiponectin leads to the persistence of the body’s anti-inflammatory response, which may result in an excessive inflammatory reaction, potentially triggering sepsis. Conversely, an increase in adiponectin contributes to the resolution of inflammation, aligning with the observed elevation of adiponectin levels in patients recovering from sepsis (21). Therefore, the mechanisms by which adiponectin regulates adaptive immune responses may also play a role in the immune dysregulation observed in sepsis.

## 5 Adiponectin prevents organ damage in sepsis

### 5.1 Endothelium

The endothelium plays a pivotal role in maintaining vascular homeostasis, and its impairment due to trauma and sepsis can result in microvascular dysfunction and subsequent organ damage (76).

Vidula Vachharajani et al. found that adiponectin deficiency is associated with increased leukocyte/platelet adhesion and blood brain barrier dysfunction in sepsis, and studies have shown that adiponectin regulates blood brain barrier dysfunction via leukocyte and platelet through an E-selectin dependent mechanism in the cerebral microcirculation during sepsis (77).

The study conducted by Hou Yun et al. revealed that treatment with adiponectin demonstrated a mitigating effect on endothelial cell apoptosis and exerted a protective influence. Adiponectin intervention exhibited its capability to delay endothelial cell apoptosis in vascular tissue by downregulating plasma malondialdehyde (MDA) levels in mice (78). To further elucidate the protective mechanism of adiponectin against endothelial cell injury, the researchers investigated the oxidative and endoplasmic reticulum (ER) stress mechanisms in human umbilical vein endothelial cells (HUVECs). The findings confirmed that adiponectin effectively attenuated endothelial cell apoptosis by suppressing endoplasmic reticulum stress, suggesting its potential as a therapeutic strategy for safeguarding endothelial cells and thus preventing sepsis-induced coagulation dysfunction (78). Adiponectin has previously demonstrated its potential to attenuate hypercoagulability by inhibiting oxidative stress-induced injury and apoptosis in endothelial cells of septic mice (78, 79).

The presence of adiponectin can alleviate endothelial dysfunction and inhibit endothelial injury, thereby improving coagulation dysfunction and reducing tissue damage. These findings present potential for early therapeutic intervention in clinical settings aimed at preventing coagulation dysfunction resulting from sepsis.

### 5.2 Liver

The rapamycin (mTOR) signaling, known as rapamycin-sensitive pathway, plays a pivotal role in regulating the immune cell-mediated inflammatory response. Suppression of mTOR activity effectively attenuates the production of proinflammatory cytokines, thereby exerting an inhibitory effect on inflammatory signals (80). The potential therapeutic effects of adiponectin on sepsis-induced hepatocyte injury have been investigated in recent studies, focusing on its interaction with AMP-activated protein kinase (AMPK) within the mechanistic target of mTOR signaling. Results indicate that administration of adiponectin effectively reduces inflammatory factor levels in rats compared to controls, indicating its capacity to inhibit mTOR signaling via AMPK activation and subsequently alleviate hepatocyte apoptosis and liver injury during sepsis (15).

The administration of adiponectin can exert a hepatoprotective effect by attenuating hepatic inflammation (81). It can mitigate hepatocyte apoptosis through the inhibition of pro-inflammatory factor secretion.

### 5.3 Heart

The presence of endotoxin in the bloodstream plays a pivotal role in initiating septic shock. Various proinflammatory cytokines, such as TNF-α and IL-6, can induce cardiac dysfunction during sepsis and subsequently lead to heart failure (82). Yoshio Watanabe et al. investigated the impact of adiponectin on LPS-induced acute cardiac injury *in vivo*. In adiponectin-knockout mice, there was an increase in TNF-α and IL-6 expression following LPS induction, which could be attenuated by etanercept (a soluble TNF receptor), resulting in improved left ventricular systolic function; furthermore, administration of adenoviral vector expressing adiponectin (Ad-APN) ameliorated LPS-induced left ventricular dysfunction in adiponectin-deficient mice, accompanied by a reduction in TNF-α and IL-6 expression (83). These findings suggest that adiponectin exerts a protective effect against LPS-induced acute cardiac injury by suppressing the inflammatory mediators TNF-α and IL-6, thereby attenuating cardiac inflammation.

The dephosphorylation of cardiac connexin 43 (Cx43) is associated with myocardial apoptosis and fibrosis, and is observed in arrhythmias, ischemic myocardial injury, as well as heart failure (84, 85). The sepsis model illustrates that adiponectin exerts regulatory control over the expression of proinflammatory cytokines TNF-α and IL-6, while concurrently attenuating myocardial inflammation and ameliorating dysfunction through modulation of Cx43 expression, thus effectively averting myocardial injury (85). The findings from this study demonstrated that Cx43 expression was upregulated in mice with suppurative sepsis, whereas adiponectin downregulated Cx43 expression, providing evidence that adiponectin could attenuate LPS-induced apoptosis by suppressing Cx43 expression and protect against LPS-induced myocardial injury through activation of the PI3K/AKT signaling pathway. These results suggest that adiponectin may represent a promising therapeutic approach for managing sepsis-related myocardial injury (86).

The regulation of gap junction proteins by adiponectin can attenuate myocardial injury, while the inhibition of proinflammatory cytokine secretion can also contribute to the reduction of myocardial injury.

### 5.4 Lung

Recent research findings indicate that adiponectin exhibits anti-inflammatory activity in chronic obstructive pulmonary disease, asthma, and invasive fungal infections. The presence of adiponectin receptors in the lung suggests a significant role for adiponectin in various biological processes within the lungs (13).

Adiponectin, an anti-inflammatory adipokine, facilitated the reduction of TNF-α levels in the lungs of mice infected with *Mycobacterium tuberculosis* (87).The administration of adiponectin mitigated the pulmonary injury induced by LPS in the ALI model (88). The Knockout of adiponectin (APN KO) mice with invasive aspergillosis demonstrate enhanced disease pathology, including decreased survival rates, elevated pulmonary fungal burden, augmented cytokine production (IL-6 and TNF-α), and heightened eosinophil recruitment (49).

Adiponectin improves lung damage in various lung diseases, suggesting that targeting enhanced signal transduction could be a potential therapeutic approach. However, further investigation is still required to elucidate the protective pathways mediated by adiponectin in fungal, bacterial, and viral infections associated with lung diseases. These findings may pave the way for novel preventive strategies against infection, hyperinflammation, and other pulmonary pathologies.

## 6 Adiponectin level changes did not align with the expected theoretical pattern

At present, it is generally believed that adiponectin is an anti-inflammatory factor. As a protective factor, the change of adiponectin level is theoretically positively correlated with a good prognosis of the disease. As described above, adiponectin is an anti-inflammatory factor, and the decrease of adiponectin may cause the transition inflammation of the infected body and trigger sepsis. The increase of adiponectin level is beneficial to the recovery of immune homeostasis in patients with sepsis. In contrast, persistently low levels encourage transitional inflammation to continue, leading to disease exacerbation and even death. However, conflicting results have been reported in other investigations.

For instance, a prospective survival prediction study examined 170 critically ill patients (122 with sepsis and 48 without sepsis) admitted to the intensive care unit (ICU), comparing them with a healthy control group of 60 individuals. The patients’ survival rate was monitored for approximately three years. Those critically ill patients who presented a serum adiponectin level below the cut-off value upon ICU admission exhibited higher short-term survival rates and overall three-year survival rates compared to those exceeding the cut-off value (89). This result was also reported in Ingeborg D Welters et al. The study conducted by revealed that the adiponectin levels were significantly elevated in patients with septic shock compared to those with sepsis, and similarly, the adiponectin levels were higher in non-survivors of sepsis than in survivors (p<0.001) (21). According to the data provided by Andreas Hillenbrand et al., it was observed that adiponectin levels in survivors were significantly lower on day 1 after onset compared to before onset. Conversely, analysis of data from deceased patients revealed a slight increase in adiponectin levels on the first day after onset when compared with premorbid levels (22).

These data demonstrate an elevation in adiponectin levels among patients with severe shock and subsequent mortality following sepsis, thereby seemingly contradicting the prevailing theory of adiponectin as a protective and anti-inflammatory mechanism. However, it is important to consider that patients with sepsis may exhibit varying disease stages, wherein inflammation and immunosuppression can manifest either sequentially or concurrently (90–93). The assessment of the probability of a poor prognosis cannot solely rely on the testing of a single parameter before or after sepsis onset, nor can it be based solely on independent analysis of samples from surviving and deceased patients.

For instance, the study cohort conducted by Andreas Hillenbrand et al., consisting of 22 patients, revealed a slightly lower median plasma adiponectin concentration 1 day after the onset of sepsis compared to the concentration observed prior to sepsis onset (22). This could potentially be attributed to the inadequate anti-inflammatory effects exerted by low levels of adiponectin during the pre-sepsis inflammatory phase, thereby resulting in Hyperinflammation and subsequent progression towards sepsis. The study, however, in independent analyses of sepsis patients who survived and those who died, the deceased individuals exhibited higher concentrations of adiponectin (22). This phenomenon may arise due to the body’s post-sepsis response of enhancing adiponectin secretion as a means to counteract the systemic inflammatory reaction. However, in cases where immune regulation is imbalanced, sustained anti-inflammatory effects can lead to persistent immune suppression, exacerbating patient condition and potentially resulting in fatality. Therefore, adiponectin exhibits distinct alterations in various pathological conditions and exerts diverse prognostic effects at different stages of disease progression.

In the early stage, prompt elimination of pathogens by the immune system facilitates rapid restoration of immune balance (90). This process may involve a transition from low to high levels of the anti-inflammatory factor adiponectin, as increased adiponectin levels can play an anti-inflammatory role to control excessive inflammation and regulate immune homeostasis (18, 94). However, if the pathogen is not promptly eliminated, the regulation of adiponectin secretion becomes compromised in patients with metabolic disorders, leading to sustained elevation of adiponectin levels and consequent long-lasting anti-inflammatory response. This persistent anti-inflammatory milieu fosters chronic immunosuppression (91). Consequently, these individuals become susceptible to secondary infections, resulting in prolonged immunosuppression, immune collapse, and potential physical disability. This condition is commonly referred to as persistent inflammation immune-suppression catabolism syndrome (90). Severe immunosuppression ultimately culminates in shock or mortality (92). This observation is consistent with the clinical findings of Alexander Koch et al. and Ingeborg D Welters et al., which critically ill and deceased patients demonstrated elevated levels of adiponectin. This may elucidate the elevated adiponectin levels observed in patients experiencing shock or mortality. This just shows that adiponectin plays an anti-inflammatory role. However, persistently high concentrations may elicit an exaggerated anti-inflammatory response, leading to immunosuppression and ultimately contributing to a poor prognosis in septic patients.

In conclusion, during the early stage of sepsis, patients exhibit a hyperinflammatory state characterized by decreased levels of adiponectin. In the intermediate stage, elevated adiponectin levels contribute to immune homeostasis restoration, however, prolonged and continuous high levels may serve as a predictor for adverse outcomes in later stages. Therefore, dynamic monitoring of adiponectin at different stages of sepsis is crucial for understanding the immune status and holds significant implications for disease prevention and treatment.

The prediction of mortality has limitations, as changes in adipokine levels may not be solely attributed to sepsis but also to the surgical procedure itself in certain subjects. Considering the interindividual variations among patients, aberrant liver and kidney function may exert an influence on adiponectin levels, thereby contributing to metabolic irregularities (81). For certain patients with diabetes or obesity, a notable decrease in circulating adiponectin levels may be observed (95, 96). However, in obese individuals with sepsis, augmented adipose tissue may compensate by upregulating the production and secretion of anti-inflammatory mediators such as adiponectin (97), thereby potentially facilitating the resolution of excessive inflammation during sepsis. Nevertheless, if there is an early robust response of adiponectin followed by incomplete metabolic recovery, it could lead to further immune dysfunction and consequently result in an unfavorable outcome (98). Refractory immunosuppression facilitates the emergence of secondary infections, which have been linked to subsequent mortality (99). Diabetic patients are prone to experiencing glucose and lipid metabolic disorders, which can subsequently result in aberrant production of inflammatory cytokines, immune response dysregulation, delayed pathophysiological damage to the body, and heightened mortality rates following infection (100). These inter-individual variations may give rise to contradictory findings.

Additionally, the expression or secretion of adiponectin may be influenced by certain medications following treatment. The administration of statins may potentially modulate the equilibrium between proinflammatory and anti-inflammatory adipokines (101). Katherine Robinson et al. hypothesized that the regulation of adiponectin in sepsis patients could potentially benefit from the impact of statins. The preliminary study unveiled, for the first time, a strong correlation between changes in adiponectin levels and statin usage among sepsis patients (102). Michael Behnes et al. investigated the alterations in adiponectin expression in cultured human adipocytes under an inflammation model and in patients with severe sepsis following activated DAA administration. The research findings suggest a potential association between adiponectin and the pathogenesis of systemic inflammatory response in sepsis, wherein DAA treatment can induce upregulation of adiponectin expression (103).

In conclusion, critically ill and dying patients with septic shock exhibit elevated levels of adiponectin. The underlying mechanism for this phenomenon may involve the role of adiponectin in the pathological process leading to death. However, other variables such as abnormal tissue and organ function, individual metabolism, or metabolic disorders caused by dysfunction during early critical illness may also contribute to this elevation. Therefore, to mitigate the impact of these confounding factors, it is imperative to closely monitor the basal level of patients and track the fluctuations in adiponectin levels throughout the sepsis stage, rather than solely relying on a “reference range” for assessing sepsis status and patient condition.

## 5 Summary and prospects

Adiponectin exerts an anti-inflammatory effect, and its levels fluctuate in accordance with the severity of sepsis; However, the underlying cause for this phenomenon remains unestablished. The modulation of hyperinflammation and the protective impact on tissue cells can be attributed to adiponectin’s distinctive characteristics in septic conditions. This review elucidates the mechanisms underlying adiponectin alterations during sepsis.

There remains an urgent imperative to develop biomarkers for the clinical management of sepsis. Adiponectin, as a factor implicated in the onset and progression of sepsis, holds promise as a marker reflecting immune response and organ dysfunction within personalized diagnostic models and precision medicine approaches. This would enable a more comprehensive understanding of the sepsis phenotype, enhance diagnostic accuracy, and guide medication decisions. Further experimental and clinical investigations are warranted to elucidate the pathophysiological role of adiponectin in critical illness and ascertain its suitability as a biomarker or supplementary scoring system for predicting survival rates among critically ill patients.

Although adiponectin has been utilized for prognostic purposes in sepsis, its association with obesity, insulin resistance, diabetes, and inflammatory diseases implies that the clinical trajectories and outcomes of sepsis patients with these underlying conditions may vary significantly. Therefore, the utilization of adiponectin as a biomarker is subject to certain limitations and necessitates adjustment for confounding factors during subject analysis. In future studies, it would be beneficial to differentiate changes in adiponectin levels among sepsis patients with different underlying diseases to facilitate targeted therapy. Another limitation in the clinical management of sepsis lies in its rapid progression through various stages accompanied by diverse outcome manifestations. Investigating the dynamics of adiponectin throughout the entire process of sepsis represents an appealing avenue for providing more comprehensive evidence supporting clinical diagnosis and treatment.

The mechanism of adiponectin in sepsis remains unconfirmed, and the pathogenesis and progression of the disease are intricate, thus no studies have been conducted on utilizing adiponectin as a therapeutic agent for sepsis. In future research endeavors aimed at developing adiponectin as a potential treatment for sepsis, it is crucial to consider its mechanisms and effects across diverse populations with varying underlying diseases and different stages of the condition.
